# Cholangiocarcinoma identified in perforated choledochal cyst in a 3-year-old boy

**DOI:** 10.1186/s12887-024-04709-y

**Published:** 2024-04-05

**Authors:** Lun Yu, Wenli Xiu, Aimei Yue, Xiwei Hao, Zhong Jiang, Jie Wu, Qian Dong

**Affiliations:** 1 Department of Paediatric Surgery, The Affiliated Hospital of Qingdao University, Qingdao, 266000 China; 2 Department of Paediatrics, The Affiliated Hospital of Qingdao University, Qingdao, 266000 China; 3 Department of Pathology, The Affiliated Hospital of Qingdao University, Qingdao, 266000 China

**Keywords:** Choledochal cyst, Surgery, Three-dimensional, Cholangiocarcinoma, Child

## Abstract

Cholangiocarcinoma in patients with Choledochal cysts is rare in childhood; however, it seriously affects the prognosis of the disease. The key to addressing this situation lies in completely removing the extrahepatic cyst. We herein present a case report of a 3-year-old boy with cholangiocarcinoma associated with a choledochal cyst (CDC). Preoperative 3D simulation, based on CT data, played an important role in the treatment of this patient.

## Introduction

Choledochal cyst (CDC) is a common biliary abnormality in East Asian populations, with an estimated incidence of approximately 1 in 1000 births [1]. Typical symptoms at presentation include abdominal pain, palpable abdominal mass and jaundice. A few asymptomatic cases have been detected during imaging tests, such as abdominal sonography and computed tomography. When possible, prompt complete surgical excision and biliary tract reconstruction are the mainstays of treatment, for preventing long-term complications such as liver failure and malignancy [2].

Spontaneous bile duct perforation and cholangiocarcinoma are both relatively rare but severe conditions in CDC patients. Among perforated CDC cases, 42.1% occurred in infants; however, only 1.5% occurred in children aged > 10 years [3]. One-stage surgery was considered difficult to perform in this situation because of the presence of severe inflammation and the potential disruption of the anastomosis. However, evidence constantly shows that one-stage surgery is a safe procedure if the patient is stable [4]. Similarly, the incidence of cholangiocarcinoma according to the CDC is also age-related, as approximately 30 to 40% of cases occur in patients aged older than 50 years and 0.4% of cases occur in children [5].

Due to the low incidence of this disease, there have been limited reports of cholangiocarcinoma with CDC in young children. Iwai et al. reported on a case of adenocarcinoma in a choledochal cyst of a 12-year-old girl [6]. Tanaka et al. described a case of advanced cholangiocarcinoma in an 11-year-old male patient [7]. Saikusa et al. reported on a 3-year-old boy with invasive cholangiocarcinoma [8]. We herein report a 3-year-old patient who unfortunately had perforation and cholangiocarcinoma at the same time. To the best of our knowledge, this case represents the youngest patient ever documented in this particular scenario, and this patient shares the same age as the youngest CDC patient with cholangiocarcinoma ever reported in the world [8].

## Case report

A 3-year-old boy was admitted to the hospital due to recurrent abdominal pain and vomiting for three days. The abdomen was not distended, but the right upper quadrant was tense, with fixed tenderness and rebound tenderness. The patient had no history of previous abdominal surgery. Laboratory tests revealed an elevated white blood cell count of 17.98 × 10^9^/L, a C-reactive protein level of 22.91 mg/L, a procalcitonin level of 0.065 ng/mL, and a blood amylase concentration of 286 U/L. The patient was started on intravenous fluids, cefoperazone sulbactam, cimetidine, and somatostatin. Subsequently, meropenem was administered as an upgraded antibiotic due to the exacerbation of abdominal pain and increase in the level of C-reactive protein (63.64 mg/L).

**Fig. 1:**
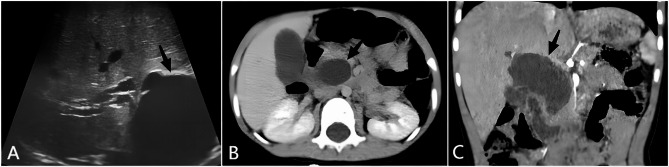
Routine imaging studies before surgery. A: Abdominal ultrasonography revealed cystic dilatation of the common bile duct measuring 6.3 × 3.7 × 3.1 cm (arrow); B: CT transverse section view of the CDC surrounded by the pancreas (arrow); C: CT coronal section view of the CDC (arrow)

**Fig. 2:**
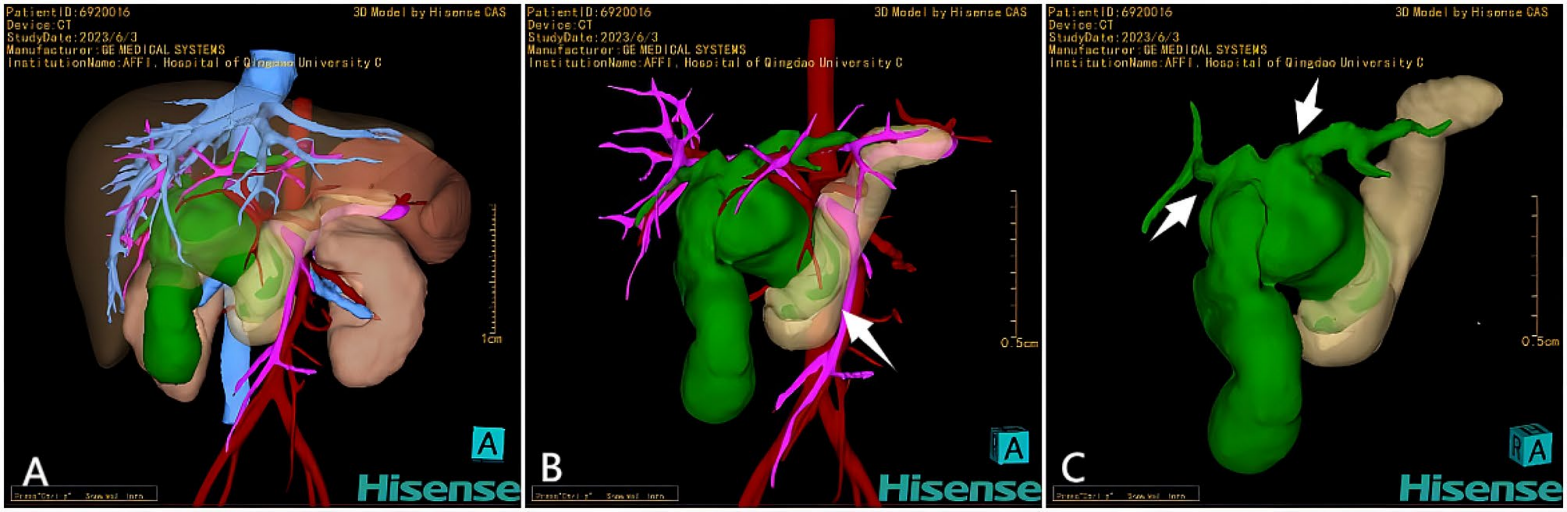
3D reconstruction via the Hisense computer-assisted system. A: Dynamic appearance and surrounding structures of choledochal cysts and gallbladder (depicted in green). B: Through the functions of translucency of the pancreas and transparency of the liver, a CDC was visualized in close proximity to the artery (depicted in red) and portal vein (depicted in pink) and the pancreas (depicted in yellow). The lower pole of the CDC is surrounded by pancreatic tissue (arrow). C: The openings of the left and right hepatic ducts were spatially distant according to the 3D image (arrows)

Abdominal ultrasonography revealed cystic dilatation of the common bile duct measuring 6.3 cm×3.7 cm×3.1 cm, accompanied by intrahepatic bile duct dilatation and enlargement of the gallbladder. Contrast-enhanced computed tomography also revealed cystic dilatation of the common bile duct and slight dilatation of the intrahepatic bile duct, while no enhancement was observed in the cystic wall (Fig. 1). The CT data were reconstructed using the Hisense computer-assisted surgery (Hisense CAS) system to generate a dynamic three-dimensional (3D) image (Fig. 2). Using this system, the surgeons can assess the dimensions of the cyst and adjacent organs and the orientation of the extrahepatic bile duct and can develop a surgical plan if necessary.

**Fig. 3:**
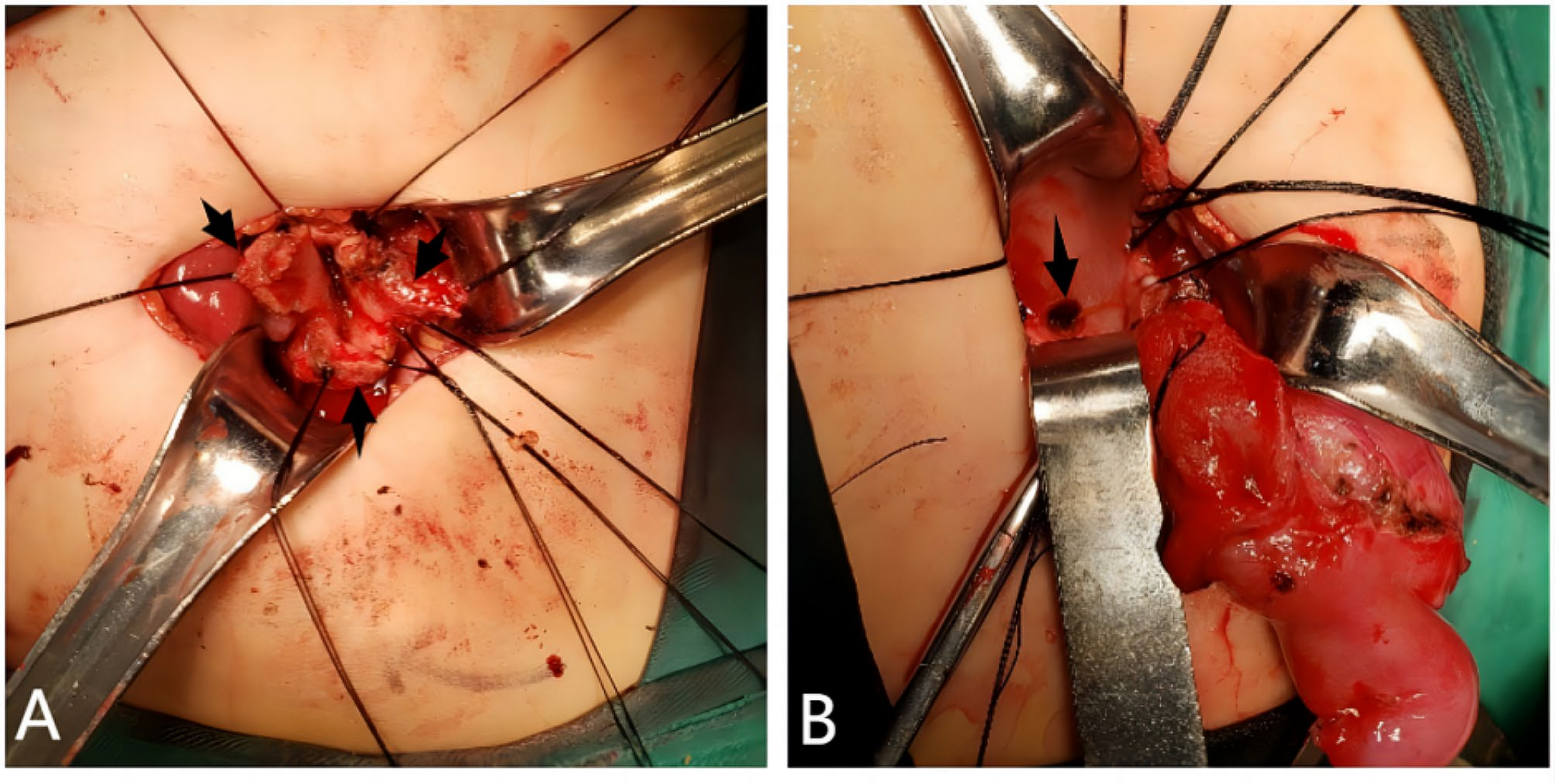
Intraoperative findings of the case. A: The wall of the bile duct exhibited oedema and indurations (arrows). B: A large perforation was observed in the posterior wall of the cyst after the cyst was opened (arrow)

After one week of conservative treatment, we reevaluated the patient. The white blood cell count decreased to 14.23 × 10^9^/L on laboratory testing, and the C-reactive protein level decreased to 36.86 mg/L. Notably, liver function tests revealed elevated serum aspartate aminotransferase (1372 U/L), alanine aminotransferase (1096 U/L), and bilirubin (38.6 µmol/L) levels, which were within the normal range upon admission. Surgery was performed on the tenth day of hospitalization (Fig. 3). The wall of the bile duct exhibited oedema and indurations, and the bile duct was also adhered to the greater omentum. The surrounding tissue was highly susceptible to haemorrhage during separation of the cyst. After the cyst was opened, a large perforation was observed in the posterior wall. The common bile duct was excised smoothly from the hepatic hilum to the junction of the pancreatic duct. In accordance with the 3D image, the openings of the left and right hepatic ducts were spatially distant, so the plasty of left and right hepatic ducts was performed. Conventionally, the technique of hepaticojejunostomy involves Roux-en-Y anastomosis.

**Fig. 4:**
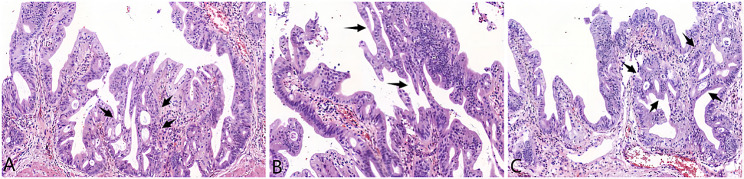
Histology of the cholangiocarcinoma in the case (HE×100). A-C: The glandular structures were ethmoid or multilayered in cancerous sites (arrows). Interglandular fusion was observed. Epithelial cells also had typical atypia

The patient made a smooth recovery and was discharged from the hospital 7 days postsurgery. The postoperative pathology revealed that the cyst wall consisted of proliferating fibrous tissue and was lined by biliary epithelium, consistent with a diagnosis of choledochal cyst. Some areas of the epithelium exhibited high-grade biliary intraepithelial neoplasia, while glandular structures in certain regions appeared disordered, indicative of malignancy. Notably, the cancerous tissue was confined to the mucosal layer without evidence of metastasis in the examined peripheral lymph nodes (Fig. 4). The ductal resection margins were negative.

The patient did not experience any complications during the initial one-month or six-month postoperative follow-up period. Paediatric hematologists and pathologists participated in an outpatient consultation, where it was determined that chemotherapy was currently unnecessary and that close monitoring was advised.

## Discussion

Spontaneous bile duct perforation in some patients can potentially be fatal due to the young age of many patients and the presence of nonspecific symptoms, such as fever and abdominal distention. An analysis on 133 children who experienced CDC perforation revealed that the mean age at the time of surgery was 1.37 years (2 days to 12.37 years) [9]. However, if treatment is delayed, bile peritonitis may develop, which is characterized by aseptic chemical inflammation and relatively mild clinical manifestations compared to bacterial peritonitis. Despite advancements in imaging techniques, such as abdominal sonography, contrast-enhanced computed tomography, magnetic resonance cholangiopancreatography, and hepatobiliary scintigraphy, preoperative identification of the perforation site is rare [10, 11]. Most of the time, surgeons judge the timing of perforation based on the general condition of the child, the effect of conservative treatment, and the physical signs of the abdomen.

Considering the progressive increase in C-reactive protein and transaminase levels posttreatment in this patient, along with persistent indications of peritonitis, we hypothesized that biliary obstruction and perforation occurred. The patient’s overall condition was satisfactory, demonstrating haemodynamic stability prior to surgery, possibly due to rapid omental encapsulation of the tissue adjacent to the perforation.

Additionally, the application of Hisense CAS system also increases the confidence of surgeons. By analysing the CT data, this system enables noninvasive examination and facilitates rapid and precise generation of 3D images. Moreover, it allows for adjustment of viewing angles, spatial rotation, and selective concealment of nonrelevant organs to accurately locate the anatomical structures. Previous studies have suggested that Hisense CAS system can visually and stereoscopically display the anatomical variations of hepatic vessels and hilar bile ducts prior to surgery [12, 13]. Therefore, this system is widely used to treat hepatobiliary system diseases at our our center [14–16]. In this case, we utilized the system to accurately quantify the cyst and gallbladder volumes, identify the correlation between the distal end of the common bile duct and pancreatic tissue as well as the variation in the openings of the left and right hepatic ducts, and exclude the presence of adjacent encapsulated abscesses or alterations in the right hepatic artery. It may be crucial to gather comprehensive information prior to one-stage surgery when confronted with possible inflammatory adhesion, bleeding, oedema, and indistinct layers. The junction of the pancreatic and bile ducts, as well as the common channel, failed to be displayed in enhanced CT or Hisense CAS system, possibly due to the thinness of the proximal pancreatic duct. We suppose that magnetic resonance cholangiopancreatography (MRCP) may be a better examination for diagnosing pancreaticobiliary maljunction (PBM). However, due to the severity of acute abdomen on admission and low compliance of this patient, we did not complete MRCP for further evaluation.

The risk of carcinogenesis in childhood CDC has not been given enough attention in the past, but in theory, the risk is always present. CDC is thought to occur in the process of the development of PBM [17]. In PBM patients, the pancreatic and bile ducts join at a site that lies outside the area of influence of the papillary sphincter, and this phenomenon leads to the reciprocal reflux of pancreatic juices and bile, as well as the activation of pancreatic enzymes [18]. The mutation of genes gradually occurs in where there is repeated damage and repair of the bile duct mucosa [19]. A study conducted by Mori et al. revealed high expression of carcinogenesis-related genes, such as KRAS and p53, in the biliary epithelium of paediatric patients with CDC, mirroring the expression patterns observed in adults with PBM. The result of this study may indicate that CDC in children possesses a comparable carcinogenic potential to PBM in adults [20]. Ono et al. also reported a significantly greater Ki-67 labelling index in the gallbladder mucosa of children with CDC than in that of controls, suggesting that mucosal changes could be initiated beginning in early childhood [21]. In a study involving 210 children with CDC, epithelial premalignant lesions, including intestinal metaplasia, pyloric gland metaplasia, and low-grade dysplasia, were identified in 78(37.1%) patients [22].

For patients with CDC, a malignancy may be suggested by the presence of an intracystic mass on imaging. However, similar to this case, a small intramural cancer often remains undetectable and can be detected by microscopy alone [23]. Complete excision of extrahepatic cysts is recommended for the treatment of CDC as the standard surgical approach due to the high risk of carcinogenesis associated with incomplete removal [24]. For CDC patients with cholangiocarcinoma, we recommend individualized treatment according to the Union for International Cancer Control (UICC) staging guideline [25]. The stage of cholangiocarcinoma in this patient is pTisN0M0 (stage 0) because tumour is confined to the mucosal layer, without extension to the muscle layer or fibrous tissue. It is rare for cholangiocarcinoma to be diagnosed as carcinoma in situ (CIS) [26]. Previous studies have noted that the depth of tumor invasion is closely associated with prognosis for cholangiocarcinoma [27, 28]. However, there were very few research on the treatment and prognosis of cholangiocarcinoma in situ. Whether adjuvant chemotherapy should be used in patients at stage 0, especially for children, remains questionable. Radical surgery is supposed to be the only curative-intent treatment. Although cholangiocarcinoma in our case was at a very early stage, we noticed that the presence of bile duct perforation may increase the risk of bile extravasation and tumor dissemination. Thus, it is crucial to maintain a rigorous and continuous follow-up.

In conclusion, the management of CDC diagnosed in childhood necessitates prompt intervention to mitigate the potential risk of carcinogenesis. Notably, the presence of perforation does not preclude the completion of the one-stage operation.

## Data Availability

The datasets used and/or analyzed during the current study are available from the corresponding author upon reasonable request.
